# Spotlight on cGAS-STING: role in disease pathogenesis and therapeutic potential

**DOI:** 10.1186/s43556-026-00478-5

**Published:** 2026-06-05

**Authors:** Dan Yan, Jiaoyue Hu, Zuguo Liu, Weijie Ouyang

**Affiliations:** 1 Department of Ophthalmology, The Affiliated Hospital of Guizhou Medical University, Guizhou Medical University, Guizhou, Guizhou Province China; 2 Xiamen University affiliated Xiamen Eye Center, Fujian Provincial Key Laboratory of Ophthalmology and Visual Science, Fujian Engineering and Research Center of Eye Regenerative Medicine, Eye Institute of Xiamen University, School of Medicine, Xiamen University, Xiamen, Fujian China; 3 Changsha Aier Eye Hospital, Changsha, Hunan Province China; 4 Department of Ophthalmology, Xiang’an Hospital of Xiamen University, Xiamen, Fujian Province China; 5 Department of Ophthalmology, The First Affiliated Hospital of University of South China, Hengyang, Hunan Province China

**Keywords:** cGAS–STING pathway, Innate immunity, Metabolic reprogramming, Tumor immunology, Targeted therapy

## Abstract

The cyclic GMP-AMP synthase (cGAS)-stimulator of interferon genes (STING) signaling pathway represents a cornerstone of innate immunity, functioning as the primary cytosolic DNA sensor in mammalian cells. Upon detecting pathogenic DNA or mislocalized self-DNA (such as leaked mitochondrial or micronuclear DNA), cGAS synthesizes the second messenger cGAMP, which subsequently activates endoplasmic reticulum-resident STING. This activation triggers an intricate signaling cascade involving liquid–liquid phase separation, dynamic organelle trafficking, and robust interferon and pro-inflammatory cytokine production, thereby bridging microbial defense, antitumor immunity, and cellular homeostasis. Despite these structural and functional insights, the pathway's context-dependent duality, dictating whether it activates protective acute immunity or drives pathological chronic inflammation, immunosuppression, and metabolic dysregulation, remains a critical, unresolved clinical challenge. This review systematically integrates recent breakthroughs across structural biology, nanotechnology, and clinical research to dissect the spatiotemporal dynamic regulation and non-canonical functions of the cGAS-STING axis. We comprehensively examine its cell-type-specific mechanisms and metabolic-immune crosstalk within the microenvironments of neurodegenerative diseases, oncology, and autoimmune disorders. Furthermore, we highlight emerging translational innovations, emphasizing the rational design of small molecule inhibitors, advanced nanocarrier delivery systems, and combination immunotherapies. By redefining the conventional understanding of cytosolic DNA sensing, this synthesis establishes a comprehensive roadmap for precision immunomodulation. Ultimately, it provides a crucial framework for developing next-generation, microenvironment-adaptive therapeutics that leverage spatiotemporal dynamics to treat cGAS-STING-related pathologies.

## Introduction

The cGAS–STING signaling pathway represents a cornerstone of innate immunity, evolving from a fundamental DNA-sensing mechanism into a multifaceted regulator of physiological and pathological processes [1]. Since the seminal discoveries of cGAS in 2013 and STING in 2008, this pathway has revolutionized our understanding of cytosolic nucleic acid surveillance, bridging microbial defense, cancer immunology, and autoinflammatory disorders. Its core function of detecting exogenous or endogenous double-stranded DNA to initiate interferon (IFN) and inflammatory cytokine production positions it as a critical sentinel against viral infections, cellular damage, and malignant transformation [2]. The pathway's architectural elegance, characterized by phase separation-driven cGAS activation and organelle-trafficking-mediated STING signaling, enables rapid, context-dependent immune responses. Recent technological advances, including cryo-EM structural analyses and CRISPR-based screening platforms, have unveiled unprecedented layers of complexity, revealing non-canonical functions in mitochondrial homeostasis, metabolic reprogramming, and chromatin regulation that extend beyond traditional IFN-centric paradigms.

Structurally, the cGAS–cGAMP–STING axis operates through a meticulously orchestrated cascade. The cGAS-cGAMP-STING axis is the primary dsDNA-sensing pathway in mammalian cells [3]. In homeostasis, nuclear cGAS remains suppressed via nucleosome binding, while cytoplasmic cGAS exists in an autoinhibited state. Pathogen invasion or cellular stress triggers mitochondrial/nuclear DNA leakage into the cytosol, prompting cGAS dimerization and liquid–liquid phase separation. This facilitates cyclic GMP-AMP synthesis, which binds endoplasmic reticulum-resident STING dimers [4, 5]. Subsequent conformational changes enable STING translocation to the Golgi apparatus, where it recruits TBK1 via its C-terminal tail domain. TBK1 undergoes trans-autophosphorylation, phosphorylating STING and IRF3 to drive IFN gene expression [6–8]. Concurrently, STING activates NF-κB through TRAF6 and IKK complexes, amplifying inflammatory responses [9, 10]. This dual-signaling architecture underpins the pathway's versatility, allowing tailored immune outcomes based on stimulus duration, subcellular localization, and tissue microenvironment.

The pathway's biological significance extends far beyond antimicrobial defense. In oncology, it exhibits Janus-faced behaviors, whereby early activation promotes antitumor immunity through dendritic cell maturation and CD8 + T cell priming, while chronic signaling induces PD-L1 upregulation and myeloid-derived suppressor cell recruitment, fostering immunosuppression [11]. Autoimmune diseases like systemic lupus erythematosus demonstrate pathological cGAS activation by self-DNA [12], while neurodegenerative conditions exhibit impaired STING trafficking [13]. Metabolic disorders further complicate this landscape, as nutrient fluctuations dynamically reshape signaling thresholds, such as when elevated Zn^2+^ potentiates cGAS activity [14]. These context-dependent phenotypes underscore the necessity of spatiotemporal precision in therapeutic targeting [15].

## Fundamental mechanisms and spatiotemporal dynamic characteristics of cGAS-STING

To fully appreciate how the cGAS-STING pathway orchestrates such diverse and context-dependent immune outcomes, it is essential to first understand its foundational mechanics. This section delves into the fundamental processes by which cGAS senses cytosolic DNA and the subsequent spatiotemporal dynamics that govern STING activation and trafficking. By mapping these core events, we can better understand how cells distinguish between physiological homeostasis and pathogenic stress.

### Mechanisms of cGAS sensing cytosolic DNA

**Fig. 1:**
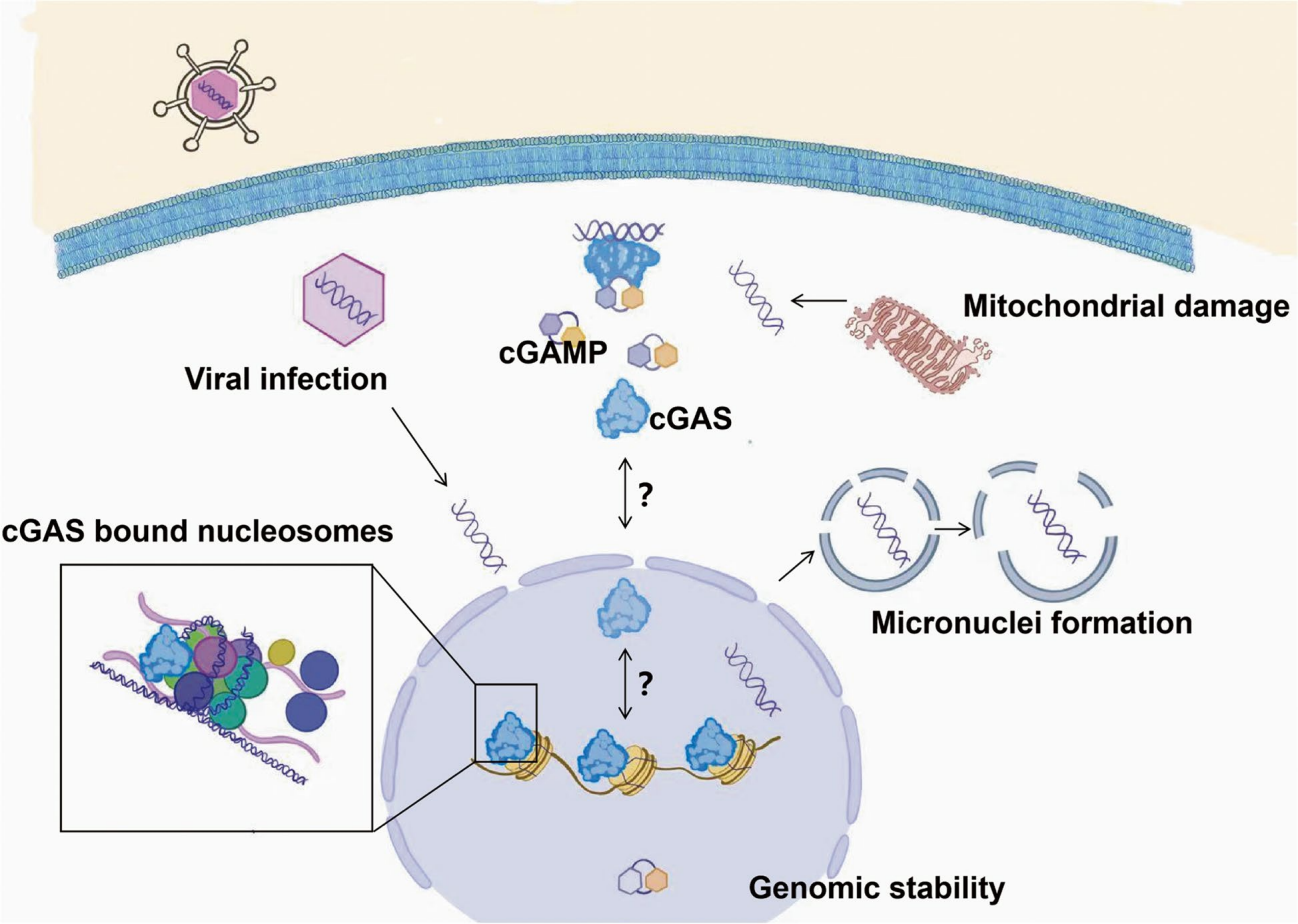
cGAS compartmentalization in homeostasis. Two cGAS pools exist intracellularly: (1) Majority sequestered in the nucleus on nucleosome acidic patches (inactive), (2) Minor cytosolic pool capable of signaling. Activation occurs via pathogenic DNA (e.g., viral) or self-DNA from mitochondrial/genomic damage. Activated cGAS produces cGAMP. Key unresolved questions include cGAS mobilization from nucleosomes, dynamic nuclear-cytosolic trafficking, and histone modification effects on nucleosome tethering. Created using Adobe Illustrator 2023

As a cytosolic DNA sensor, cGAS detects dsDNA from pathogens or self-sources (Fig. 1), catalyzing cGAMP synthesis to activate STING-mediated interferon and cytokine expression, which serves as a critical defense mechanism [16, 17]. Its specificity is modulated by DNA features, in that dsDNA longer than 45 bp strongly activates STING, while shorter fragments of 20–40 bp competitively inhibit activation [18]. This length dependence suggests that cGAS performs precise regulation in the recognition of exogenous DNA and the prevention of accidental activation of self-DNA.

In addition to the length of DNA, DNA structure/modifications and protein interactions further refine activity of cGAS. For instance, the affinity of cGAS for certain specific DNA sequences may vary depending on the secondary structure of the DNA [19], while PCBP1 (poly(rC)-binding protein 1) enhances cGAS-DNA binding and catalytic efficiency [20]. These mechanisms highlight cGAS's reliance on both physicochemical DNA properties and protein networks. During the recognition of cytoplasmic DNA, cGAS depends on its sensitivity to DNA length, structure, and modifications. This recognition mechanism provides an effective immune defense for the host and also offers important clues for understanding the pathogenesis of various diseases, especially in infections, inflammation, and autoimmune diseases where abnormal function of cGAS may lead to severe immune dysregulation [21, 22].

### STING activation and trafficking pathway

STING acts as a key immune adaptor, sensing cytosolic DNA to trigger immune responses. Its function relies on dynamic ER-to-Golgi/lysosome trafficking. cGAMP, produced by cGAS-catalyzed synthesis, activates STING and promotes its translocation from the ER to the Golgi via COPII vesicles [23]. This process depends on molecular switches like COPII assembly (Sar1/Sec23/24 complexes) and Rab GTPases (e.g., Rab1/Rab14), which regulate vesicle targeting and fusion [24, 25]. Phosphorylation events during trafficking are critical for regulating STING activity and determining the amplitude of downstream signaling [26].

### Spatiotemporal regulation of signal transduction

Subcellular localization of cGAS-STING activation determines functional outcomes. cGAS detects pathogenic/self-DNA in the cytoplasm, forming condensates that enhance activation through spatiotemporally regulated clustering [27]. This spatial control allows precise immune responses under physiological/pathological conditions.

Spatiotemporal dynamics govern signal intensity/duration. Acute cGAS-STING activation in tumor microenvironment (TME) suppresses tumorigenesis, while chronic activation may induce immunosuppression and tumor progression [28, 29]. Cross-talk with pathways like autophagy further modulates infection responses and inflammation [30]. Understanding these mechanisms provides therapeutic insights for cancer, infections, and autoimmune diseases.

## Molecular architecture and activation mechanisms of the cGAS–STING pathway

Building upon the fundamental mechanisms of DNA sensing and STING trafficking, a deeper layer of regulation dictates the precise threshold and intensity of the immune response. This regulation is intricately tied to the molecular architecture of the pathway components, their assembly dynamics, and complex post-translational modifications. In the following section, we explore how these structural and biochemical nuances meticulously fine-tune pathway activation, ranging from the formation of liquid–liquid phase-separated microreactors to the newly discovered proton channel functions of STING.

### Liquid–liquid phase separation: a "microreactor" for cGAS activation

**Fig. 2:**
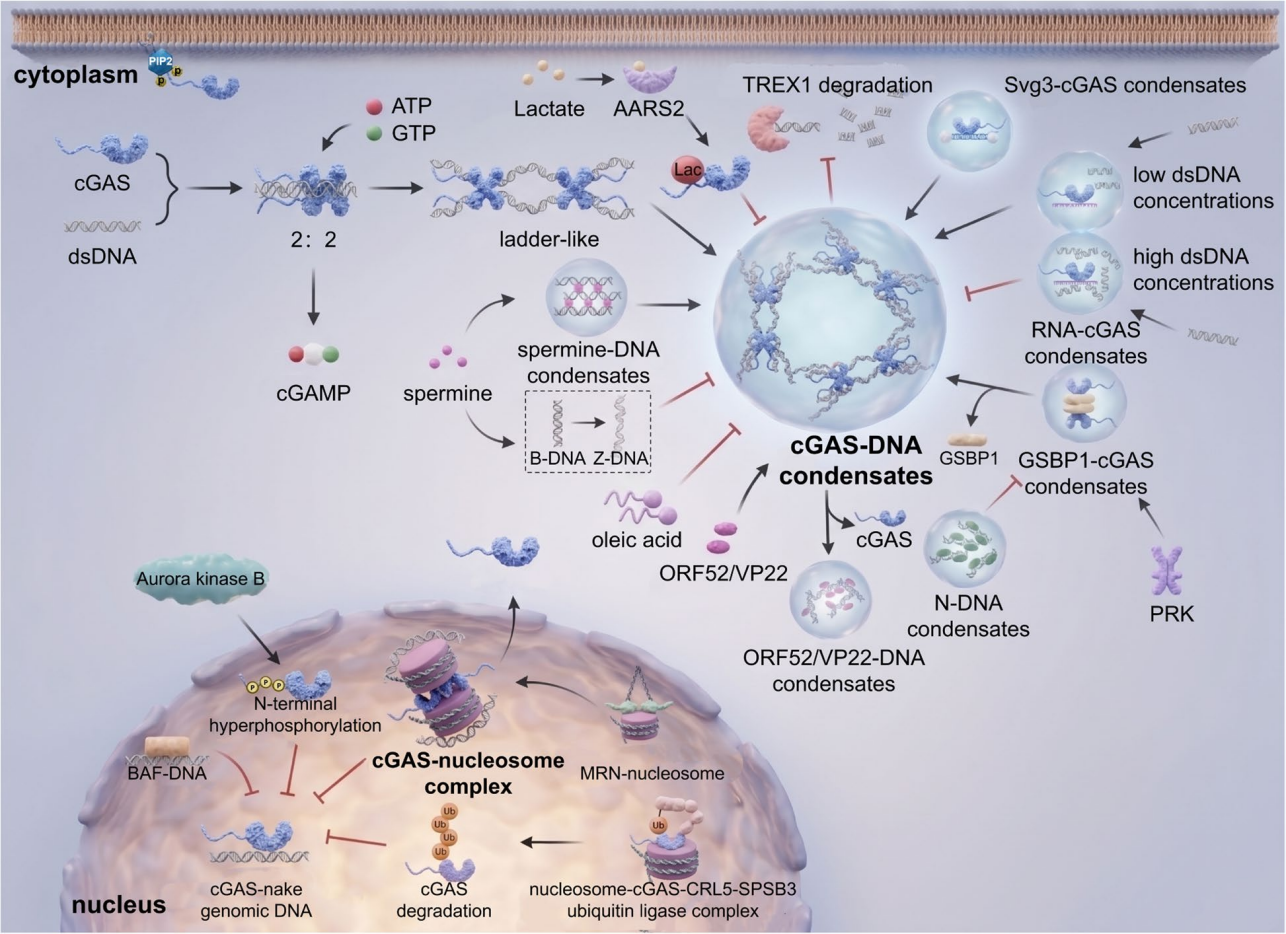
Overview of cGAS activation, inhibition, and phase-separation dynamics. In the cytoplasm, cGAS engages double-stranded DNA to form a 2:2 signaling complex that assembles into a stable ladder-like polymeric network, enabling the production of cGAMP from ATP and GTP. Multivalent cGAS–DNA interactions further promote the emergence of higher-order phase-separated condensates that amplify signaling. In contrast, nuclear cGAS is restrained through high-affinity binding to nucleosomes, which prevents productive DNA engagement and suppresses its enzymatic activation. Created using Adobe Illustrator 2023

cGAS, a critical DNA sensor, forms signaling microenvironments via LLPS to enhance its function [31] (Fig. 2). Its N-terminal disordered region binds Zn^2^⁺, inducing LLPS to form condensates that improve dsDNA recognition and catalytic activity for cGAMP synthesis [32–34]. Long DNA drives phase separation more effectively than short DNA by providing additional binding sites [33]. Physiological regulators include G3BP1, which maintains pre-phase-separated cGAS granules to accelerate LLPS efficiency; loss of G3BP1 impairs responsiveness [35]. Molecularly, cGAS dimerization and weak DNA-binding sites (A/B/C) form the activation basis, while LLPS concentrates cGAS-DNA complexes (100–1000 ×) to achieve a threshold-dependent activation response [14]. This phase separation not only protects cGAMP from enzymatic degradation through physical isolation but, more importantly, creates a high-concentration "microreactor," significantly enhancing catalytic efficiency under low DNA concentration conditions. Dynamic modulation involves Zn^2^⁺ stabilizing LLPS to enhance activity, viral proteins (e.g., HSV ORF52/VP22) disrupting condensates via phase separation, and oleic acid dissolving them to suppress immunity [14, 36, 37]. Functionally, condensates create selective environments where spatial barriers restrict TREX1 activity, protecting DNA for efficient sensing, while autoimmune-related TREX1 mutations disrupt this balance [34]. DDX3X promotes cGAS activation via intrinsic phase separation, and USP8-mediated deubiquitination enhances condensates to amplify interferon responses [38]. We believe that LLPS is not merely a physical aggregation but rather functions as a selective filtration mechanism. It selectively concentrates catalytic substrates (ATP/GTP) while physically shielding and inhibiting the approach of negative regulatory factors (such as TREX1). Future therapeutic strategies that target and intervene in this dynamic equilibrium of phase separation (for example, by altering intracellular Zn^2^⁺ concentration or using small molecules to interfere with N-terminal interactions) will provide a new perspective for the suppression of autoimmune diseases.

### STING: a paradigm shift from signal pump to proton valve

STING comprises N-terminal transmembrane domains (TMD) and C-terminal ligand-binding domains (LBD). The TMD contains α-helices that form a helical bundle in dimeric STING, anchoring it to the ER membrane [39, 40]. TMD-mediated dimerization in a head-to-head orientation fuses ER membranes, preventing STING translocation and TBK1 recruitment [39]. In 2022, Defen Lu et al. discovered that C53 binding at the TM2-TM4 interface induces TM4 displacement, driving STING oligomerization and immune activation independently of cGAMP [41]. TMD is critical for ER phase separation to regulate immune homeostasis [40] and LC3 interaction in autophagy [42]. Despite its well-established canonical signaling functions, STING was for a long time considered merely a simple adaptor protein. However, recent studies have revealed its unique role as a pH-dependent ion channel. Resting-state STING maintains a closed conformation via TM4-TM4' interlocking, while cGAMP/C53 binding triggers structural rearrangement, forming a 2.4 nm hydrophilic pore for proton transport [39, 43]. This function is particularly prominent in post-Golgi vesicles, leading to vesicle deacidification, promoting LC3 lipidation, autophagy, cell death, lysosomal pH and membrane fusion [44–47], which is independent of the function that induces IRF3 activation. This redefines its role from a conventional immune adaptor to a pivotal immune-metabolic coupling nexus.

The proton channel function of STING is a core mechanism in mediating non-canonical autophagy, inflammasome activation, and cell death, but dysregulation of this function is closely associated with various diseases. STING-associated vasculopathy with onset in infancy (SAVI) is a severe autoinflammatory disease caused by acquired functional mutations in the STING1 gene, such as N154S. Common pulmonary interstitial diseases and tissue damage in SAVI patients cannot be fully alleviated by traditional IFN receptor knockout (IFNAR^−/−^). The mutation allows STING to form ion channels even in the absence of ligands, leading to ion dysregulation and subsequent pyroptosis, which is central to driving tissue damage in SAVI. Targeting and blocking the pore region of STING (e.g., using small molecule C53) may be more effective than blocking its signaling transduction [48]. In the Krabbe disease model, STING activates the transcription factor TFEB through proton channel function, inducing the expression of lysosomal genes (such as Gla, Gpnmb), promoting lysosomal biosynthesis and damage repair [47].This dual function dictates that the design of STING inhibitors must be precise. Existing STING antagonists (such as H-151) primarily target cysteine sites, and whether they can simultaneously inhibit ion channel activity remains to be verified. Developing small molecules that can specifically block the STING pore may be a better approach for treating autoinflammatory diseases such as SAVI.

### Assembly dynamics of the STING–TBK1–IRF3 activation complex

The assembly dynamics of the STING-TBK1-IRF3 complex involve multiple key steps, primarily responding to activators like cytosolic DNA or cGAMP. Upon binding, STING translocates from the ER to the Golgi/endolysosomal compartments via autophagy-dependent mechanisms. Its C-terminal tail (CTD) acts as a scaffold to recruit TBK1 and IRF3, promoting TBK1-mediated IRF3 phosphorylation and nuclear translocation for downstream gene expression [49–52]. This process relies on STING conformational changes, such as oligomerization to stabilize complex assembly, and rapidly triggers innate immune responses after TBK1-catalyzed IRF3 phosphorylation. Regulatory mechanisms prevent overactivation, including ULK1-mediated STING phosphorylation by cGAMP to terminate activity [51]. In non-mammalian models (e.g., insects), STING uses conserved cRHIM motifs for oligomerization and adaptor binding (e.g., IMD), ensuring efficient signaling [53]. The dynamics integrate spatial localization, protein clustering, and modification events with high dynamism and conservation. STING activation forms a complex with TBK1 and IRF3, crucial for interferon gene expression. Dynamic binding, regulated by STING conformation and TBK1 activation, promotes IRF3 translocation and phosphorylation, initiating interferon expression. This provides insights into spatiotemporal regulation and therapeutic strategies [2].

### Organelle transport network regulating cGAS–STING activation

#### ER-golgi trafficking mechanisms

In resting cells, STING primarily resides in the ER. Upon activation, it translocates to the Golgi apparatus, initiating downstream signaling cascades. Trafficking relies on vesicular transport, whereby COPII mediates anterograde transport from the ER to the Golgi, while COPI handles retrograde transport [54]. Membrane bending, crucial for ER-Golgi trafficking, is regulated by STEP1, which interacts with lipids to alter membrane properties and promote vesicle formation. TAK1 phosphorylation (e.g., S355) is vital for ER-Golgi trafficking, modulating membrane dynamics and vesicle formation [55, 56]. These mechanisms provide therapeutic targets for diseases involving intracellular trafficking defects. Modulating STEP1 or TAK1 activity could intervene in STING trafficking, regulating cGAS-STING pathway activation to offer new treatment strategies.

#### Golgi-lysosome degradation pathway

STING clustering and activation at the Golgi is critical for initiating downstream immune responses. To prevent overactivation, STING is transported from the Golgi to post-Golgi endosomal structures for degradation. The Golgi-lysosome degradation axis is essential for cellular metabolism. The AP-1 complex and ARF1 protein play key roles by recognizing phosphorylated CTT (cargo transport signal) to mediate vesicle formation and transport. Specifically, AP-1 facilitates selective cargo transport, whereas ARF1 regulates membrane dynamics to support vesicle formation. Beyond the classical lysosomal pathway, ESCRT complexes also mediate STING degradation [54]. ESCRT contributes to microautophagy by degrading and recycling cellular macromolecules [56, 57]. These mechanisms reveal cellular trafficking complexity and provide new perspectives on disease pathogenesis.

#### Non-canonical nuclear functions and degradation of cGAS

cGAS localizes not only to the cytoplasm but also to the nucleus. Nuclear cGAS interacts with nucleosomes to block self-DNA binding, preventing autoimmune activation [58, 59]. It also regulates gene expression and DNA repair, such as through its interaction with Prmt5 to modulate interferon gene transcription and virus-induced cytokine production [60, 61]. cGAS interacts with DNA repair proteins like Ku complex to balance NHEJ/HR pathways, ensuring genome integrity [62, 63]. Nuclear cGAS activity is tightly regulated by cellular states, such that during senescence or inflammation, its expression and activity modulate DNA repair capacity and inflammatory intensity. This regulation impacts age-associated chronic inflammation and related diseases, highlighting the dual role of cGAS as both an immune regulator and a metabolic player [64, 65].

The nuclear envelope acts as a barrier and regulator for nuclear cGAS. Membrane integrity is crucial for cGAS localization and activity. Nuclear envelope rupture exposes DNA to the cytoplasm, activating cGAS-STING. cGAS degradation at the nuclear membrane involves interactions with nuclear membrane proteins [66]. Studies in cardiomyopathy/cancer models show nuclear envelope damage activates cGAS, triggering abnormal immune responses [67, 68].

Nuclear cGAS degradation primarily depends on UPS. SPSB3, as a substrate receptor for CRL5 E3 ligase, ubiquitinates cGAS to promote its degradation [69]. This maintains immune homeostasis; cGAS overactivation may cause autoimmunity. During cell division, cGAS degradation prevents nonspecific genomic activation [70]. The NLS of cGAS influences nuclear entry, stability, and activity, though this relationship requires further investigation [59].

When the degradation mechanism of cGAS is disrupted, it can lead to excessive accumulation of cGAS, causing the body to produce self-antibodies and leading to the occurrence of autoimmune diseases. For example, studies have shown that the absence of SPSB3 leads to abnormal accumulation of nuclear cGAS, which in turn triggers enhanced type I interferon (IFN-I) signaling, particularly significant in infectious or autoimmune diseases [69]. Additionally, overactivation of cGAS can cause genomic instability and promote tumor development under certain circumstances [71]. Furthermore, abnormal accumulation of cGAS within cells is associated with the onset of various inflammatory diseases. Research has found that DNA damage within cells during certain disease states can activate cGAS, triggering downstream inflammatory responses. If these responses cannot be regulated through normal degradation mechanisms, they may result in chronic inflammation and tissue damage [72]. Therefore, research into the cGAS degradation pathway can help us understand the pathogenesis of autoimmune diseases and potentially provide potential targets for new treatment strategies. By regulating the degradation of cGAS, it may assist in restoring immunological homeostasis and reducing the risk of autoimmune diseases.

### Post-translational modifications and immune regulation in cGAS-STING pathway

#### PTMs of cGAS

Strict regulation of cGAS activity via post-translational modifications (PTMs) is essential for immune homeostasis. PTMs, including phosphorylation, ubiquitination, SUMOylation, acetylation, and methylation, critically control cGAS stability, enzymatic activity, and ligand-binding capacity [73–76]. Phosphorylation/dephosphorylation governs cGAS subcellular dynamics. B-lymphoid tyrosine kinase phosphorylates Y215 to maintain cytoplasmic localization in resting cells, while immune DNA triggers dephosphorylation and nuclear translocation [77]. Akt phosphorylates S305 (S291 in mice), inhibiting ATP/GTP binding and reducing cGAMP/IFN-β production, increasing HSV-1 infectivity [78]. Protein phosphatase 6 dephosphorylates S435/S420 in resting cells but dissociates during DNA virus infection, promoting GTP-binding and enzymatic activity [20]. CDK1-cyclin B phosphorylates S305 to suppress cGAMP synthesis in mitosis, reversible by PP1 [79]. Aurora kinase B hyperphosphorylates N-terminal sites during mitosis, blocking chromatin DNA binding; PP1/PP2A mediate dephosphorylation post-mitosis [73–76, 80].

Ubiquitination is another key regulator. E3 ligases (TRIM56, RNF185) and deubiquitinases modulate ubiquitination to control stability/activity [81]. TRIM56-mediated K335 ubiquitination enhances dimerization, DNA binding, and antiviral responses [82]. RNF185 catalyzes K27-linked polyubiquitination at K137/K384, boosting enzymatic activity [83]. K48-linked polyubiquitination may target cGAS for autophagic degradation (mechanism unclear). Post-infection, TRIM14 recruits USP14 to cleave K48 chains, preventing degradation [84, 85]. USP27X/USP29 stabilize cGAS via deubiquitination [86, 87].

SUMOylation also regulates cGAS. SUMO conjugation at K335/K372/K382 inhibits DNA binding, oligomerization, and activity [88], reversed by SENP7-mediated deSUMOylation. TRIM38 mediates SUMOylation at K464/K217 in uninfected/early-infected cells to block ubiquitin-mediated degradation, while SENP2 promotes proteasomal degradation via deSUMOylation in late infection [89].

Acetylation and methylation are critical. Aspirin-induced K384/K394/K414 acetylation inhibits activation [90], while KAT5-mediated N-terminal K47/K56/K62/K83 acetylation enhances DNA binding and antiviral immunity [91]. Protein arginine methyltransferase 5-catalyzed R124 methylation suppresses DNA binding and antiviral responses [92].

#### PTMs of STING

Similar to cGAS, STING stability, localization, and activation are regulated by PTMs including ubiquitination, SUMOylation, phosphorylation, and palmitoylation. As a downstream effector of cGAS, STING activation is highly dependent on palmitoylation. Golgi-localized palmitoylation is essential for STING activation [93]. This modification is specifically inhibited by H-151 and C-176, which covalently modify C91 to block palmitoylation-dependent STING aggregation, inhibiting TBK1 phosphorylation and IFN-I signaling. 4-Octyl itaconate (4-OI) alkylates Cys91, suppressing palmitoylation and oligomerization, thereby blocking pathway activation [94]. 2-Bromopalmitate (2-BP) inhibits palmitoylation, eliminating IFN-I responses [95]. Notably, Cys88/Cys91 palmitoylation mediates interaction with VDAC2. In renal cell carcinoma (RCC) models, 2-BP inhibition of ZDHHC palmitoyltransferases significantly impedes RCC growth [96]. Thus, palmitoylation in the cGAS-STING pathway is a key regulatory node. Inhibition of this modification (e.g., via 4-OI or 2-BP) mitigates diseases like RCC, providing a therapeutic foundation [97].

Ubiquitination plays a pivotal role in STING regulation. K63-linked ubiquitination at K224 is required for TBK1-IRF3 activation [98], a process suppressed by PCV2 virus via USP21-mediated phosphorylation, weakening antiviral immunity [99]. Bacterial c-di-GMP promotes K63-linked ubiquitination, enhancing STING stability in intestinal immunity [100]. E3 ligase TRIM10 catalyzes K27/K29-linked ubiquitination to positively regulate the pathway [101], while UFL1 inhibits ubiquitination at K338/K347/K370 to maintain stability [102]. Recent studies also reveal STING degradation via ESCRT-mediated ubiquitination [103], offering new therapeutic targets. Further research will elucidate precise ubiquitination mechanisms in the cGAS-STING pathway.

SUMOylation and phosphorylation are critical for STING stability and activation. Early in activation, STING oligomerizes to recruit and activate TBK1 (autophosphorylation). TBK1 phosphorylates S366 as a docking site for IRF3 interaction and subsequent phosphorylation [104, 105].TRIM38 mediates K338 ubiquitin-like modification of STING via chaperone-mediated autophagy (CMA), inhibiting the degradation of STING. TBK1 is transported to the late endosome/lysosomal compartment, activating transcription factors IRF3 and NF-κB. Subsequently, cGAMP activates ULK1/2 kinases, resulting in the phosphorylation of STING and a decrease in its activity at position S365 [51]. The phosphorylated STING is then transferred to peri-nuclear microsomes, recruiting SENP2 to remove STING from K337 and promoting its degradation through the CMA pathway. Conversely, TBK1 is a stronger STING kinase than ULK1, as it only phosphorylates IRF3 at position S396, marking the activation of IRF3 [106].

Furthermore, ISGylation modification at K289 promotes oligomerization of STING, enhancing its signaling capacity and further driving downstream antiviral responses [2]. Therefore, research on post-translational modifications of STING provides new insights into its role in immune responses and may offer new targets for related disease treatments.

In summary, although the PTMs regulating the cGAS/STING signaling pathway have not been fully elucidated, understanding these regulatory mechanisms may provide potential targets for drug development against cGAS/STING-related diseases.

## Role of cGAS-STING in the pathogenesis of specific diseases

While the precise structural and biochemical regulatory networks described above are critical for maintaining immune homeostasis, their dysregulation invariably leads to severe clinical consequences. Moving from molecular mechanisms to macroscopic pathophysiology, the cGAS-STING axis emerges as a pivotal driver in a wide spectrum of human disorders. This section systematically examines how the aberrant activation, or suppression, of this pathway contributes to the specific pathogenesis of neurodegenerative diseases, tumor development, metabolic syndromes, and autoimmune conditions.

### cGAS-STING pathway in neurodegenerative diseases

**Fig. 3:**
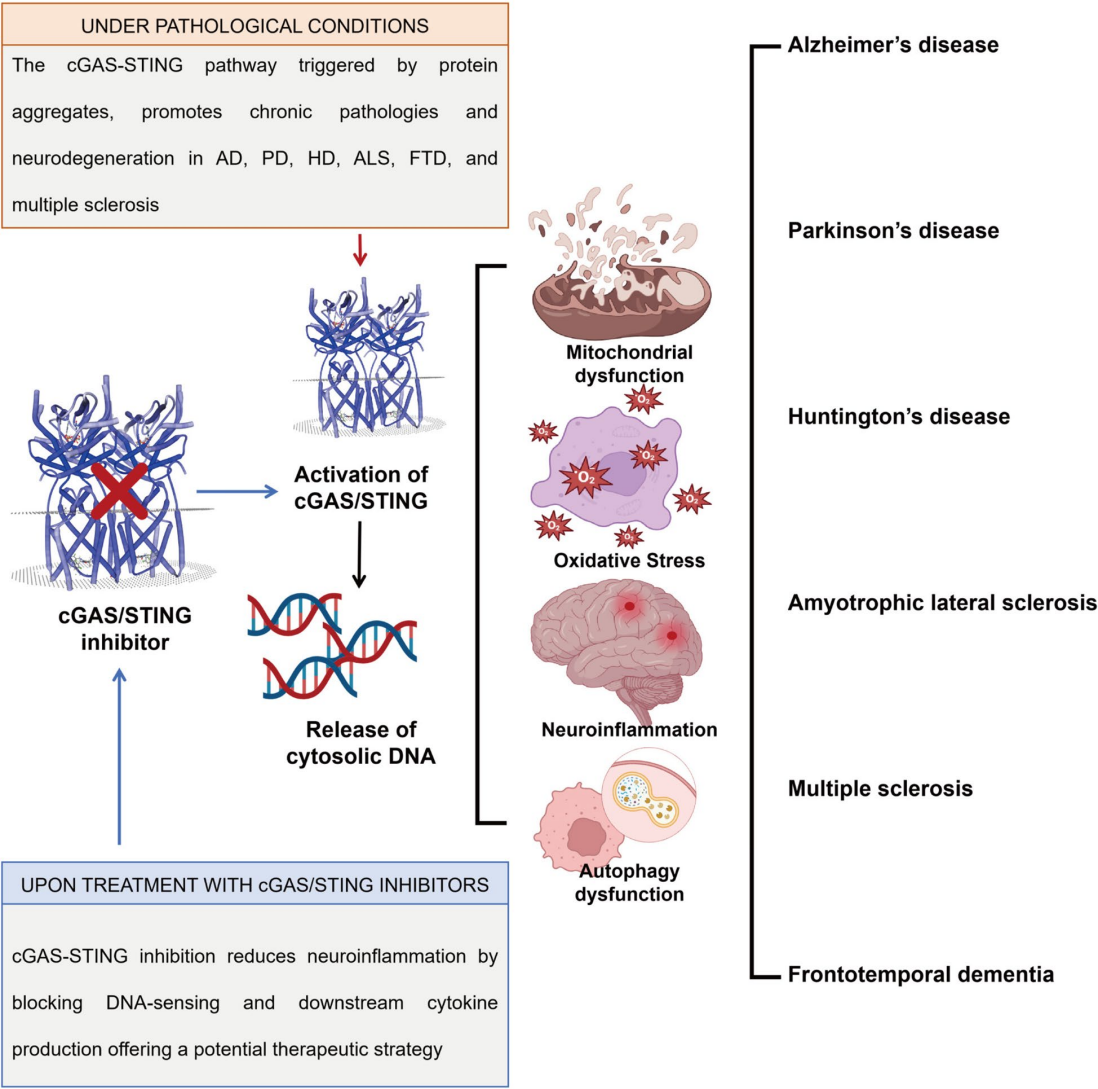
cGAS-STING pathway in neurodegenerative diseases. The cGAS-STING pathway, activated by protein aggregates, drives chronic neuroinflammation and neurodegeneration in Alzheimer's disease, Parkinson's disease, Huntington's disease, amyotrophic lateral sclerosis, frontotemporal dementia, and multiple sclerosis. Pathological activation occurs through DNA-sensing mechanisms, triggering downstream cytokine production and sustaining neuroinflammatory cascades. Therapeutic inhibition of cGAS-STING signaling mitigates neuroinflammation by blocking DNA detection and subsequent cytokine release, offering a promising strategy to attenuate disease progression. Created using Adobe Illustrator 2023

Pathological processes in NDs, such as Alzheimer's disease and Parkinson's disease, often involve mitochondrial dysfunction, leading to mitochondrial DNA leakage into the cytosol [107, 108]. This mtDNA leakage activates the cGAS-STING pathway, triggering robust inflammatory responses that are central to neuroinflammation and degeneration [13] (Fig. 3).

#### Pathological role in specific neurodegenerative diseases

##### Alzheimer's disease

AD features memory loss and cognitive decline with Aβ plaques and neuroinflammation. Recent years have witnessed growing interest in the cGAS-STING signaling pathway as a pivotal immune response mechanism implicated in the pathogenesis of Alzheimer's disease (AD). cGAS detects cytosolic dsDNA, activating STING to induce IFN-I and neuroinflammation [109]. Ferecskó et al. demonstrated a marked upregulation of STING expression by 100% in neurons and endothelial cells derived from the central nervous system tissues of AD patients [110], with increased phosphorylation of STING/TBK1/p65/IRF3 in prefrontal cortex [109]. In 5xFAD mice, immunohistochemistry showed phosphorylated STING and IRF3 colocalized with microglial marker CD68 around Aβ plaques in the dentate gyrus, indicating microglial cGAS-STING activation. cGAS ablation suppressed neurotoxic astrocyte development. Aβ-induced neurotoxicity was partially reduced by conditioned media from cGAS^−/−^astrocytes. These findings suggest the cGAS-STING axis modulates neuroinflammation through microglia-astrocyte-neuron communication [109]. Pathogenesis starts with Aβ plaques and hyperphosphorylation of tau protein, causing mitochondrial dysfunction, DNA damage, and mtDNA leakage [111–114]. Cytosolic mtDNA activates cGAS-STING, triggering NF-κB/IRF pathways in microglia/neurons, releasing TNF-α/IL-6/IFN-I [115–117]. In addition, tau proteins interact with PQBP1, a sensor of cGAS-dependent innate immunity against HIV-1 by binding reverse-transcribed HIV-1 DNA and cGAS. In microglia, extrinsic tau is recognized by PQBP1, activating cGAS-STING and inducing NF-κB nuclear translocation in vitro. Microglia-specific Pqbp1 deletion in vivo abolished PQBP1-tau colocalization, NF-κB translocation, and cGAS recruitment, reduced inflammatory gene expression, and rescued cognitive deficits in an acute tau injection model [118]. The sustained inflammatory milieu exacerbates synaptic loss and synaptic dysfunction, further promoting a vicious cycle of Aβ and tau pathology. Ultimately, these changes lead to widespread neuronal apoptosis in the hippocampus and cortex, along with cognitive decline [119, 120]. Further investigations revealed that genetic ablation of cGAS in 5 × FAD mice alleviates cognitive deficits, Aβ aggregation, neuroinflammation, and cholinergic neuronal damage. Moreover, pharmacological inhibition of STING has been shown to consistently ameliorate AD-related pathological alterations [121]. Collectively, these findings underscore the central role of the cGAS–STING signaling pathway in the progression of Alzheimer’s disease.

##### Parkinson's disease

In-depth investigations into the pathogenesis of PD have progressively elucidated the pivotal role of the cGAS-STING signaling pathway in its pathological progression. Within PD’s pathological framework, the accumulation of α-synuclein (α-syn) oligomers triggers the collapse of mitochondrial quality control and dysfunction of Parkin protein, resulting in the release of fragmented mitochondrial DNA into the cytosol, which directly activates the cGAS-STING pathway [122]. Overactivation of this pathway facilitates the binding of STING to the transcription factor YY1, impeding its nuclear translocation and consequently upregulating the expression of lipocalin-2 (LCN2). This cascade induces astrocyte senescence and inflammatory responses, whereby senescent astrocytes secrete proinflammatory cytokines such as IL-1β and TNF-α. These processes collectively establish a mitoinflammatory microenvironment that damages dopaminergic neurons in the substantia nigra, ultimately leading to motor dysfunction and neuronal loss [123]. Studies utilizing PD models have demonstrated that either deletion of the cGAS gene or administration of inhibitors (e.g., H-151) can effectively block this pathogenic cascade [124, 125]. Clinical evidence further corroborates that elevated levels of cell-free mitochondrial DNA in cerebrospinal fluid are significantly associated with the severity of motor symptoms [115, 126], underscoring cGAS-STING as a critical driver of neurodegeneration in PD. Notably, small-molecule inhibitors such as C-176 have been shown to markedly alleviate neuroinflammation and dopaminergic degeneration induced by MPTP, thereby improving motor function [127]. These findings provide novel therapeutic insights for PD, suggesting that modulation of the cGAS-STING signaling pathway holds promise for the development of innovative treatment strategies.

##### Amyotrophic Lateral Sclerosis (ALS)/Frontotemporal Dementia (FTD)

Amyotrophic lateral sclerosis (ALS) is a complex and fatal neurodegenerative disease characterized by progressive degeneration of motor neurons. In recent years, the cGAS–STING signaling pathway has been recognized to play a critical role in ALS pathogenesis, particularly in neuroinflammation and intracellular DNA sensing. Elevated levels of cGAMP, a metabolic product of cGAS signaling, have been observed in spinal cord samples from ALS patients, potentially serving as a biomarker for mitochondrial DNA release and cGAS-STING pathway activation [128]. In both ALS and frontotemporal dementia (FTD), TDP-43 proteinopathy leads to dysregulation of RNA and DNA metabolism, resulting in abnormal accumulation of nucleic acids in the cytoplasm. These nucleic acids are recognized by cGAS, which subsequently activates the STING pathway and induces overexpression of interferon-stimulated genes (ISGs) such as CXCL10 [128, 129]. This activation is particularly pronounced in motor neurons, triggering endoplasmic reticulum stress and axonal degeneration, thereby disrupting neuronal homeostasis. Concurrently, STING-dependent signaling promotes neuroinflammatory responses in glial cells, leading to the release of cytokines such as IL-6 and TNF-α, which foster a neurotoxic microenvironment that accelerates motor neuron loss [130, 131]. Studies in ALS models have further confirmed that hyperactivation of the cGAS–STING pathway can induce neuronal apoptosis [110, 132–134], underscoring its central pathogenic role in the disease.

##### Huntington's Disease (HD)

Huntington's disease (HD) is an inherited neurodegenerative disorder primarily caused by a CAG repeat expansion mutation in the Huntingtin (HTT) gene. This mutation leads to the widespread presence of mutant Huntingtin protein (mHTT) in the brain, which causes significant damage particularly in the striatum, thereby affecting motor, psychiatric, and cognitive functions. Recent studies have revealed that the pathological features of HD are closely associated with inflammatory responses. The pathogenesis begins when mHTT disrupts the autophagic clearance mechanism, while its interaction with exonuclease 1 (Exo1) and MutLα (MLH1-PMS2) impairs DNA repair pathways. Consequently, damaged mitochondria and fragmented genomic DNA accumulate in the cytoplasm of striatal neurons [135–138]. These accumulated DNA fragments activate the cGAS–STING signaling pathway, triggering NF-κB signaling in microglia and inducing the release of pro-inflammatory cytokines such as IFN-β and IL-1β. This cascade results in chronic neuroinflammation and apoptosis. Ribosome profiling has shown altered ribosomal occupancy of LC3A and LC3B, key initiators of autophagy, in HD cells. Depletion of cGAS in these cells reduces its activity, downregulates the expression of inflammatory genes, and suppresses autophagy. Conversely, restoring cGAS reactivates its function and promotes inflammatory and autophagic responses. Activated cGAS-associated micronuclei have also been detected in the cytoplasm of neurons derived from human HD stem cells [138]. These findings indicate that the cGAS–STING signaling pathway is upregulated in HD, driving both inflammation and autophagy, and suggest that targeting this pathway may offer therapeutic benefits for HD.

##### Multiple sclerosis (MS)

Multiple sclerosis (MS) is an autoimmune disorder predominantly affecting the central nervous system, leading to neuroinflammation and neuronal damage. Recent investigations into the cGAS–STING signaling pathway have provided novel insights into the pathogenesis of multiple sclerosis. Elevated STING expression has been observed in neurons of both MS patients and animal models [110, 139]. Glutamate-induced excitotoxicity triggers the activation of neuronal STING, thereby promoting neuroinflammation and neuronal cell death. This indicates that STING plays a crucial role in mediating deleterious neuroinflammatory stress responses associated with MS [140]. Administration of STING inhibitors in MS animal models significantly reduces demyelinating lesions and motor impairments [141], corroborating the indispensable role of the cGAS–STING pathway in driving neuroinflammation in MS. Conversely, several studies have reported opposing functions of the cGAS–STING signaling pathway in MS. During relapsing phases of multiple sclerosis, cGAS and STING gene expression is downregulated compared to periods of remission and healthy controls, suggesting a context-dependent neuroprotective role of the pathway [142]. Treatment with a soybean-derived serine protease inhibitor, the Bowman–Birk inhibitor (BBI), has been shown to attenuate chronic neuroinflammation in MS by activating the STING/IFN-β axis in macrophages. In healthy subjects administered oral BBI, peripheral blood mononuclear cell-derived macrophages exhibited increased production of IFN-β and IL-10 [143]. These conflicting findings underscore the complex nature of the cGAS–STING pathway in multiple sclerosis and highlight the need for further research to elucidate its precise functional mechanisms and therapeutic potential in addressing neurodegeneration and neuroinflammation associated with the disease.

#### Cell-type-specific mechanisms of cGAS-STING in neurodegeneration

##### Dysregulation of neuroimmune homeostasis

Neuroimmune dysregulation represents a central mechanism by which the cGAS-STING pathway contributes to neurodegenerative disease progression. In AD, microglia, the resident macrophages of the CNS, drive neuroinflammation through cGAS-STING activation [144–146]. These cells exert dual and context-dependent functions, in that they participate in Aβ clearance through phagocytosis and degradative enzymes while also potentially facilitating pathological spreading [147, 148]. The accumulation of Aβ induces a phenotypic shift in microglia, triggering the release of neuroinflammatory mediators and exacerbating neurotoxicity. This transition is attributed to aberrant activation of the cGAS-STING pathway in microglia. Research indicates that specific activation states of microglia, such as interferon-responsive microglia (IRM) and disease-associated microglia (DAM), play pivotal roles in AD-associated neuroinflammation [149, 150]. Modulating the activity of this pathway may help balance neuroprotective and neurotoxic effects, thereby attenuating the progression of AD.

##### Neuronal apoptosis and cognitive impairment

The mechanism by which the cGAS–STING–IRF3 signaling pathway participates in cognitive impairment through the regulation of neuronal apoptosis has been elucidated, whereby SENP7-mediated cGAS deSUMOylation activates the cGAS–STING–IRF3 signaling axis. This activation triggers microglial pyroptosis—characterized by the upregulation of GSDMD-N, Caspase1, and NLRP3 proteins along with the release of inflammatory factors such as IL-1β and IL-18—which subsequently induces hippocampal neuronal apoptosis, ultimately leading to cognitive deficits including spatial and contextual memory impairment [151]. Similar mechanisms occur in ALS and FTD, where TDP-43 pathology disturbs nucleic acid homeostasis, leading to cGAS–STING–dependent type I IFN release, NLRP3 inflammasome activation, GSDMD-mediated pyroptosis, and accelerated neurodegeneration [152].

##### Disease-specific dual-mechanism of action

Importantly, the roles of cGAS–STING are disease- and context-specific. On the one hand, its activation is frequently associated with the onset of neuroinflammation. Aberrant activation of the cGAS-STING signaling cascade induces inflammatory responses in neural cells, exacerbating neuronal injury and death [146, 153]. Pathological hyperactivation, triggered by mitochondrial DNA leakage or dysregulation of nucleic acid homeostasis, can drive chronic neuroinflammation, leading to microglial polarization toward a pro-inflammatory phenotype and the release of neurotoxic factors such as TNF-α and ROS. These events accelerate neuronal loss, a pathogenic mechanism particularly prominent in Huntington’s disease and multiple sclerosis, with pathway activation levels positively correlating with disease progression. Concurrently, cGAS-STING activation may compromise neuronal function by promoting the secretion of pathological cytokines. This impact can, in turn, modulate the pathway’s own regulation, constituting a complex feedback loop. On the other hand, the cGAS-STING signaling pathway also demonstrates significant neuroprotective functions. Research reveals its involvement not only in inflammatory regulation but also in neural regeneration and repair. For instance, activation of cGAS can facilitate the recovery of injured neural cells by modulating microglial activity, thereby supporting neuronal survival and functional restoration [154, 155]. In peripheral nerve injury repair, cGAS-STING activation enhances regenerative capacity, indicating a bidirectional regulatory role of this pathway in nervous system recovery.

Nevertheless, the dual nature and complexity of the cGAS-STING pathway render it a potential therapeutic target. A key challenge in treating neurological disorders lies in modulating this pathway to simultaneously suppress pathological inflammation and promote neuroprotection, which remains an area of active investigation. Strategies under exploration include the use of STING inhibitors to attenuate excessive inflammatory responses and harnessing cGAS activation to foster neural regeneration, representing promising directions for future research [156, 157]. The biphasic and intricate characteristics of the cGAS-STING signaling pathway underscore its significance across multiple neurological conditions. Elucidating its functional mechanisms and regulatory networks will provide novel insights for developing innovative therapeutic strategies targeting neurodegenerative and other nervous system diseases.

### cGAS-STING dichotomy in tumor immunity

**Fig. 4:**
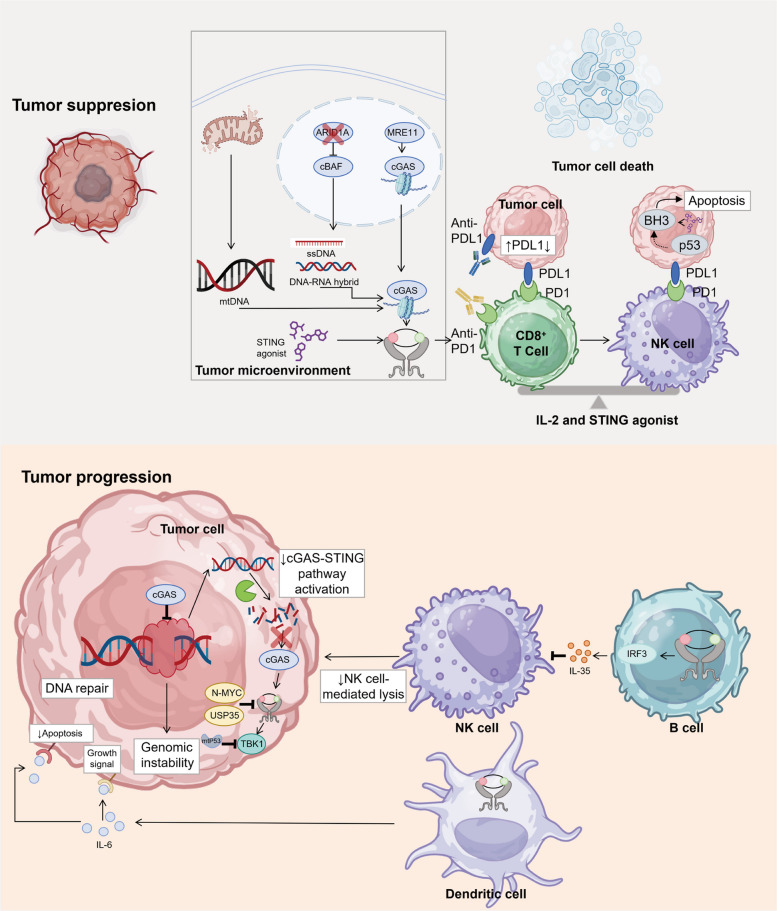
Dual roles of cGAS-STING in tumor immunology. The cGAS-STING pathway has dual roles in tumor immunology. Tumor suppression involves IFN-driven CD8⁺ T/NK cell maturation, activated by ssDNA/DNA-RNA hybrids (from ARID1A loss/cBAF inhibition), mtDNA, and MRE11-RAD50-NBN-mediated cGAS displacement for dsDNA activation. Combined IL-2/STING agonist therapy balances responses against MHC-I-deficient/sufficient tumors, while pathway activation upregulates PDL1 to enhance checkpoint blockade efficacy. STING agonists also trigger p53-independent apoptosis in p53-deficient tumors. Tumor progression results from impaired activation due to DNA repair/TREX1 overexpression, N-MYC/USP35 STING inhibition, and mutant p53 TBK1 suppression. Bregs secrete IL-35 via cGAS-STING-IRF3, inhibiting NK cells, while dendritic cells release IL-6 to transmit tumor survival signals and block IFN-JAK-STAT1-mediated apoptosis. This metabolic-immune crosstalk emphasizes context-specific therapeutic strategies. Created using Adobe Illustrator 2023

The cGAS-STING pathway exhibits a profound dichotomy within the context of tumor immunology, acting as a double-edged sword that can either suppress or promote malignancy (Fig. 4). While its acute activation is historically recognized for driving robust antitumor immunity through interferon production and T-cell priming, emerging evidence reveals that chronic, persistent signaling paradoxically fosters a pro-tumorigenic and immunosuppressive microenvironment. Understanding this delicate balance is crucial, as it dictates the trajectory of tumor progression and fundamentally influences the efficacy of cancer immunotherapies.

#### Antitumor mechanisms

Tumor cells exhibit genomic instability, oncogenic mutations, and metabolic dysregulation, causing nuclear/mtDNA leakage into cytosol (e.g., micronuclei, chromatin fragments). Subsequently, the cGAS molecules within these tumor cells recognize the DNA and generate cGAMP. Notably, the produced cGAMP can be transferred to adjacent non-tumor cells, thereby activating their STING-dependent immune responses [158–163]. Furthermore, exogenous stimuli such as chemotherapy and radiation-induced DNA exposure, oxidative stress-triggered mtDNA leakage, and mitochondrial membrane permeability alterations caused by anticancer treatments contribute to elevated concentrations of aberrant cytoplasmic DNA, facilitating rapid cGAS activation [164, 165]. Finally, DNA derived from apoptotic cells, exosomal DNA, and transposable elements have also been demonstrated to induce activation of the cGAS–STING signaling pathway in tumor cells [166, 167].

The cGAS–STING signaling pathway plays a critical role in tumor immunity. Particularly under acute activation conditions, this pathway effectively promotes IFN-I production, thereby enhancing antitumor immune responses. Within the TME, the generation and release of cGAMP can activate dendritic cells (DCs) and augment the antitumor immune response of CD8⁺ T cells. Dendritic cells serve as key regulators in antitumor immunity by capturing tumor antigens and presenting them to T cells, thereby initiating specific immune responses. Studies indicate that tumor-derived cGAMP activates DCs via the cGAS–STING pathway, promoting their differentiation into antigen-presenting cells and enhancing T cell activation and proliferation [29, 168, 169]. This process not only increases the number of tumor-specific T cells but also enhances their cytotoxicity, enabling efficient recognition and elimination of tumor cells. The positive regulatory effects of acute inflammatory responses on the TME should not be overlooked. In contrast to chronic inflammation, acute inflammation promotes remodeling of the TME, enhances immune cell activity, and suppresses tumor immune evasion. Research shows that acutely activated cGAS–STING signaling is closely associated with increased apoptosis of tumor cells and elevated expression of immune-related genes, thereby providing robust support for antitumor immunity. Moreover, acute activation of the cGAS–STING pathway is closely linked to interactions with cancer-associated fibroblasts and endothelial cells, improving tumor vascularization and oxygenation status, and creating more favorable conditions for immune cell infiltration [168, 170]. The combined use of IL-2 and STING agonists has been shown to significantly enhance the cytotoxic capacity of natural killer (NK) cells, particularly in MHC-I-deficient tumor cells, demonstrating considerable therapeutic potential. By activating the STING pathway, IL-2 not only promotes NK cell proliferation and activation but also augments their cytokine secretion capacity, thereby counteracting tumor immune evasion mechanisms [2, 171]. These findings provide new insights for cancer immunotherapy and underscore the pivotal role of the cGAS–STING signaling pathway in antitumor immunity.

#### Pro-tumor mechanisms

Chronic cGAS-STING activation in the TME promotes immunosuppression and tumor progression. For example, DMBA (a carcinogen) activates cGAS-STING, inducing DNA breaks and skin tumor formation [172, 173]. Furthermore, inactivation of the breast cancer susceptibility gene 2 (BRCA2) results in impaired DNA repair and micronucleus accumulation, thereby activating the cGAS-STING signaling pathway, which leads to cell cycle arrest, elevated interferon signaling, and cell death. Nevertheless, during chronic BRCA2 inactivation and the resulting persistent cGAS stimulation, interferon-stimulated genes (ISGs) are not only upregulated but also restore cell cycle progression, facilitating the survival of mutated cells [174, 175]. Studies indicate that sustained cGAS-STING signaling upregulates PD-L1, a key immune checkpoint molecule that suppresses T-cell activity and thereby promotes tumor immune escape [176, 177]. Research has also shown that nuclear cGAS activation can inhibit homologous recombination repair, leading to genomic instability and ultimately supporting tumor initiation and progression.

Additionally, STING activation may contribute to the formation of an immunosuppressive microenvironment. For example, regulatory T cell (Treg) activation is another important outcome of chronic cGAS-STING signaling. Increased Treg populations further inhibit effector T-cell function and reduce their capacity to attack tumor cells, thereby creating favorable conditions for tumor growth and metastasis [168]. Upon STING activation, regulatory B cells (Bregs) secrete IL-35, a cytokine that impairs natural killer (NK) cell function and diminishes their tumor-killing ability. Furthermore, alterations in the TME also play a critical role in this process. Studies reveal that interactions between cancer-associated fibroblasts and immune cells can further potentiate chronic cGAS-STING activation, resulting in sustained immunosuppression and tumor progression. Such interactions not only compromise immune cell function but may also enhance tumor cell resistance to conventional therapies, including chemotherapy and immunotherapy [170]. In the TME, cGAS-STING signaling promotes cancer metastasis via paracrine intercellular communication. Protocadherin 7, expressed in breast and lung cancer cells, facilitates the assembly of gap junctions composed of connexin 43 (CX43) between cancer cells and astrocytes, enabling cGAMP transfer from cancer cells to astrocytes. Subsequent STING activation in astrocytes triggers the secretion of TNF-α and IFN-α, which in turn activate NF-κB and STAT1 signaling in cancer cells, ultimately supporting the survival and growth of metastatic cancer cells in the brain [178]. Other studies suggest that membrane fusion-mediated STING activation, such as that occurring during increased exosome shedding from cancer cells, may also promote cancer metastasis [179]. These dual mechanisms highlight the tumor-promoting characteristics of the cGAS-STING pathway in the TME, underscoring the need for careful consideration of potential adverse effects when designing therapeutic strategies targeting STING.

#### Metabolic reprogramming in TME

Tumor metabolic reprogramming exhibits dual regulation of cGAS-STING signaling. Research indicates that cancer cells often suppress cGAS–STING pathway activity through metabolic states such as glycolysis and hypoxia. For instance, hypoxic conditions induce JNK1/2-mediated phosphorylation of PCK1, which subsequently inhibits GTP-dependent activation of cGAS, thereby facilitating tumor immune evasion [180]. Additionally, upregulation of glycolysis has been shown to limit the accumulation of cytoplasmic double-stranded DNA by increasing TREX2 expression, further inhibiting cGAS–STING pathway activation [181]. This metabolic reprogramming not only influences cancer cell survival and proliferation but also significantly impacts immune cell function within the TME.

Conversely, modulation of tumor metabolites can also activate the cGAS–STING signaling pathway. For example, studies have demonstrated that altering lactate metabolism can effectively reverse the immunosuppressive TME and enhance anti-tumor immune responses. Specific bio-mineralized outer membrane vesicles (OMVs@MnCaP-FA) have been developed to concurrently activate the cGAS–STING pathway and regulate lactate metabolism, thereby promoting anti-tumor immunotherapy [182]. This regulatory mechanism highlights the intricate interplay between tumor metabolic status and immune regulation as a critical determinant of tumor progression and therapeutic response. Furthermore, alterations in metabolic status affect not only cancer cells but also immune cells in the TME. In hepatocellular carcinoma (HCC), dysregulated lipid metabolism has been closely associated with neutrophil extracellular trap (NET) release, a process intimately linked to cGAS–STING pathway activation, underscoring the potential role of metabolic reprogramming in shaping tumor immune responses [183].

In summary, tumor metabolic reprogramming plays a pivotal role in tumorigenesis, progression, and immune escape by either suppressing or activating the cGAS–STING pathway. A deeper understanding of the interplay between metabolism and immunity may inform the development of novel therapeutic strategies aimed at optimizing anti-tumor immune responses and improving clinical outcomes.

### cGAS-STING signaling pathway: metabolic basis and disease implications

#### Metabolic regulation of cGAS-STING signaling

Recent studies indicate that aberrant activation of the cGAS-STING pathway is closely associated with metabolic disorders such as obesity, non-alcoholic fatty liver disease (NAFLD), and diabetes. Metabolites critically modulate cGAS-STING signaling via TCA cycle intermediates (e.g., citrate), amino acids (glutamate/tyrosine), and lipids [184–187]. Within the tricarboxylic acid cycle, citrate and its derivatives have been found to regulate the activity of the cGAS-STING pathway. Specifically, citrate, a key intermediate in energy metabolism, influences the cellular redox state, thereby indirectly affecting the activation mechanism of cGAS [186]. Furthermore, amino acid metabolism, particularly involving specific amino acids such as glutamate and tyrosine, has been demonstrated to impact immune responses and metabolic functions through modulation of the cGAS-STING signaling pathway [185]. Lipid metabolism also significantly influences the cGAS-STING signaling pathway; abnormal lipid accumulation and metabolic dysregulation often lead to its overactivation, contributing to chronic inflammation and metabolic syndrome [186, 187].

The relationship between redox homeostasis and signaling intensity warrants further attention. Research indicates that the cellular redox state modulates the activation of the cGAS-STING pathway, with increased oxidative stress potentially leading to excessive cGAS activation and subsequent intracellular inflammatory responses [188]. For example, in certain metabolic diseases, disruption of intracellular redox balance results in aberrant activation of the cGAS-STING pathway, exacerbating pathological progression [15]. Therefore, maintaining intracellular redox equilibrium is critical for regulating the activity of the cGAS-STING signaling pathway.

In summary, metabolites regulate the cGAS-STING signaling pathway through multiple mechanisms, involving not only changes in metabolite levels but also alterations in the intracellular redox state. Future research should focus on elucidating the precise mechanisms by which these metabolites influence the cGAS-STING pathway under various physiological and pathological conditions, thereby providing novel insights and therapeutic strategies for metabolic diseases.

#### Metabolic regulation of cGAS-STING spatiotemporal dynamics

The spatiotemporal activation of the cGAS-STING pathway (encompassing cGAS phase separation and STING ER-to-Golgi trafficking) is tightly coupled to the intracellular metabolic milieu. Specific metabolites act as critical rheostats that dynamically tune these spatial events. For instance, metal ions such as Zn^2^⁺ are essential for cGAS liquid–liquid phase separation (LLPS). Elevated cytosolic Zn^2^⁺ concentrations stabilize cGAS-DNA condensates, significantly lowering the activation threshold and boosting cGAMP production even at low DNA concentrations [14, 32]. Conversely, lipid metabolism intricately modulates these spatiotemporal dynamics. Distinct fatty acids exert opposing effects, in that while certain lipid species enhance membrane-associated STING clustering, monounsaturated fatty acids such as oleic acid can physically dissolve cGAS-DNA condensates, suppressing immune surveillance in lipid-rich microenvironments [37]. Furthermore, the spatiotemporal trafficking of STING is strictly dependent on metabolic modifications, particularly palmitoylation. Reactive lipid species, such as nitro-fatty acids, can covalently modify STING at specific cysteine residues (e.g., Cys88/91), physically impeding its transport from the ER to the Golgi apparatus and thus halting the signaling cascade [189]. Reactive oxygen species (ROS) also serve as crucial spatiotemporal regulators; oxidative stress not only triggers the leakage of mitochondrial DNA into the cytosol but also alters the redox state of STING, promoting its oligomerization and subsequent vesicular transport [188]. Together, these findings underscore that the spatial assembly and intracellular trafficking of cGAS and STING are dynamically gated by real-time metabolic fluctuations, providing a mechanistic basis for how metabolic disorders can trigger aberrant pathway localization and activation.

#### Metabolic dysregulation and disease pathogenesis

Metabolic dysregulation is closely associated with the development of numerous diseases, particularly through its impact on the cGAS–STING signaling pathway. cGAS and STING are key innate immune sensors that detect intracellular DNA to initiate immune activation. However, metabolic imbalance can lead to aberrant activation or suppression of the cGAS–STING pathway, thereby linking this signaling axis to the pathogenesis of various metabolic disorders.

Accumulating evidence indicates that the cGAS–STING pathway plays a pivotal role in multiple metabolic diseases. In conditions such as obesity, non-alcoholic fatty liver disease (NAFLD), insulin resistance, and cardiovascular diseases, excessive activation of cGAS–STING signaling is strongly implicated in chronic low-grade inflammation [190]. Energy imbalance, endoplasmic reticulum stress, and mitochondrial stress in metabolic and immune cells can all trigger cGAS–STING activation, driving pro-inflammatory responses and contributing to metabolic dysfunction [187].

In metabolic disorders such as obesity and diabetes, aberrant activation of the cGAS–STING pathway promotes the release of inflammatory cytokines, including interferons and other pro-inflammatory mediators, which further exacerbate insulin resistance and lipid accumulation, forming a self-perpetuating cycle [183, 184]. For example, studies in cardiovascular disease have shown that metabolic risk factors activate the cGAS-STING pathway through mitochondrial DNA release, nuclear DNA leakage, and ER stress, leading to chronic sterile inflammation, excessive autophagy, increased cellular senescence, and apoptosis. These processes may ultimately contribute to disease onset and progression.

Furthermore, interactions between the cGAS–STING pathway and metabolic networks play an essential role in coordinating metabolism and inflammation. The involvement of cGAS and STING in lipid metabolism is gaining increasing attention, with dysregulation of these molecules in adipocytes, hepatocytes, and renal tubular epithelial cells being linked to metabolic dysfunction and disruption of energy homeostasis. This intersection between metabolic and immune regulation suggests that targeting the cGAS–STING pathway may represent a promising therapeutic strategy for metabolic diseases.

In summary, metabolic imbalance contributes to the pathogenesis of numerous metabolic disorders by aberrantly activating or inhibiting the cGAS–STING signaling pathway. Future research should aim to unravel the complex interplay between cGAS–STING signaling and metabolic dysregulation, which may provide new mechanistic insights and therapeutic opportunities for the treatment of metabolic diseases.

### Autoinflammatory and autoimmune diseases

An increasing body of evidence demonstrates that the cGAS–STING signaling pathway is aberrantly activated across multiple autoimmune diseases (AIDs), including rheumatoid arthritis (RA), aicardi–Goutières syndrome (AGS), and systemic Lupus Erythematosus (SLE). Conversely, pharmacological or genetic inhibition of this pathway markedly reduces the production of IFN-I and other pro-inflammatory cytokines, underscoring its central role in driving autoimmune pathogenesis [191]. In this section, we summarize the regulatory functions and pathogenic implications of the cGAS–STING pathway across representative AIDs.

#### Systemic lupus erythematosus (SLE)

SLE is characterized by multisystem inflammation that leads to widespread tissue damage. The overall mortality of patients with SLE is approximately 2–3 times higher than that of the general population, with particularly elevated risk among women, ethnic minorities, and individuals aged ≥ 65 years [12, 192]. IFN-I are widely recognized as central pathogenic drivers in SLE, acting as potent activators of both innate and adaptive immune responses. Recent studies have demonstrated that the cGAS–STING pathway, as a key cytosolic DNA-sensing mechanism, plays a critical role in initiating IFN responses through the recognition of nuclear or mitochondrial DNA. This pathway has emerged as an essential contributor to SLE onset and disease progression [193]. For example, An et al. reported significantly elevated cGAS mRNA levels in peripheral blood mononuclear cells (PBMCs) from SLE patients, with expression positively correlated with anti-dsDNA antibody titers and overall disease activity scores [193]. Additionally, Willemsen et al. showed that genetic deletion of cGAS suppresses IFN production, decreases inflammatory cell infiltration, and alleviates joint swelling, highlighting the causative role of this pathway in lupus-related inflammation [193].

#### Psoriasis

Psoriasis is a chronic systemic inflammatory disease frequently accompanied by multiple comorbidities, including cardiovascular disorders, psoriatic arthritis, non-alcoholic fatty liver disease, metabolic syndrome, and psychiatric conditions such as anxiety and depression [194]. In Europe and North America, its prevalence is approximately 2%. Pan et al. reported that STING is strongly implicated in psoriasis pathogenesis and may represent a promising therapeutic target [194]. Skin biopsies from psoriasis patients revealed marked activation of the cGAS–STING pathway, which positively correlates with IRF3 phosphorylation and inflammatory responses [195]. Conversely, inhibition of this pathway exerts potent anti-inflammatory effects and significantly reduces disease severity in vivo. Collectively, these findings suggest that pharmacological suppression of the cGAS–STING pathway may offer a novel strategy for the treatment of psoriasis.

#### Rheumatoid arthritis (RA)

RA is a chronic systemic inflammatory disorder characterized by symmetrical polyarthritis affecting the hands, wrists, knees, and feet. In recent years, accumulating evidence has highlighted the crucial involvement of the cGAS–STING signaling pathway in RA pathogenesis [193]. Wang et al. demonstrated that the expression levels of cytosolic dsDNA and cGAS correlate closely with RA severity. Both cytosolic dsDNA and cGAS were markedly elevated in fibroblast-like synoviocytes (FLS) and synovial tissues from RA patients [196]. Immune complexes formed by dsDNA and anti-dsDNA autoantibodies can activate cGAS, triggering robust IFN-I production and breaking immune tolerance, thereby sustaining chronic joint inflammation. Notably, genetic deletion of cGAS or STING in FLS significantly reduced dsDNA-induced cytokine and protease production, leading to decreased cartilage destruction. Mechanistically, this protective effect was associated with reduced phosphorylation of IRF3 and NF-κB p65. In addition, excessive activation of osteoclasts, which are key mediators of bone erosion in RA, has also been shown to involve aberrant STING upregulation, further supporting the role of this pathway in mediating RA-related joint damage [197].

#### Aicardi–Goutières syndrome (AGS)

AGS is an autosomal recessive inflammatory encephalopathy characterized by leukodystrophy, cerebral atrophy, chilblain-like skin lesions, intracranial calcifications, and markedly elevated levels of IFN-α [198]. The disease is primarily caused by mutations in genes involved in nucleic acid metabolism, including RNase H2A/B/C, SAMHD1, and TREX1 [199]. Among these, TREX1 functions as a major cytosolic exonuclease responsible for degrading mislocalized nuclear and mitochondrial DNA, and loss-of-function mutations in TREX1 are well-established drivers of AGS pathogenesis [200, 201]. Strikingly, Trex1^⁻/⁻^ develop severe systemic inflammation (particularly fulminant myocarditis) and typically die within several months after birth. However, genetic deletion of either cGAS or STING completely rescues Trex1^⁻/⁻^ mice from this lethal autoimmune phenotype, indicating that aberrant activation of the cGAS–STING pathway is indispensable for disease development. Consistently, Gray et al. demonstrated that cGAS deficiency abolishes tissue inflammation, prevents autoantibody production, and normalizes survival in Trex1⁻/⁻ mice [202]. These findings firmly position the cGAS–STING axis as a central pathogenic driver in AGS and highlight it as a highly compelling therapeutic target.

#### COVID-19 and immune-mediated diseases

Since the global outbreak of SARS-CoV-2 in late 2019, overwhelming inflammatory responses have been recognized as a key determinant of severe COVID-19 outcomes [195]. Multiple studies have demonstrated that SARS-CoV-2 activates the cGAS–STING pathway through diverse mechanisms. Neufeldt et al. reported that viral infection activates the cGAS-STING-NF-κB axis in lung epithelial cells, which are the primary site of infection, thereby inducing robust inflammatory signaling [203]. Additionally, SARS-CoV-2 spike protein–mediated cell–cell fusion leads to nuclear envelope instability and micronuclei formation, which are subsequently detected by cGAS, resulting in STING activation and amplified IFN responses [203]. Importantly, inhibition of this pathway yields protective effects. Li et al. demonstrated that pharmacological inhibition of STING significantly attenuates cytokine production and tissue damage in mice six days post-infection, highlighting its therapeutic potential against SARS-CoV-2–induced immunopathology [204].

## Therapeutic targeting of the cGAS-STING pathway

Given the extensive involvement of the cGAS-STING pathway in the diverse pathogenic mechanisms outlined above, it has naturally emerged as a highly compelling target for therapeutic intervention. Transitioning from disease biology to clinical application, this section highlights the latest advancements in the pharmacological modulation of this axis. We will review the rational design of small-molecule inhibitors and agonists, biologic therapies, drugs targeting downstream effectors, and evaluate the clinical promise of emerging combination strategies.

### Small-molecule inhibitors of the cGAS-STING pathway

Current therapeutic strategies for autoinflammatory and autoimmune diseases primarily focus on developing small-molecule inhibitors that directly target the cGAS-STING axis to block aberrant IFN-I and pro-inflammatory cytokine production [205–207]. Representative small-molecule cGAS inhibitors are summarized in Table 1, while key STING inhibitors are detailed in Table 2.

**Table 1:** Representative cGAS inhibitors

cGAS inhibitors	Structure	Inhibition mechanism	Biological effect	Refs.
Aspirin	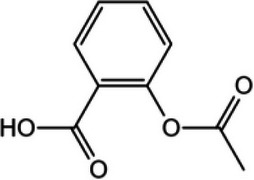	Acetylated Lys amino group of cGAS protein	Alleviates DNA-induced autoimmunity in AGS mouse models and patient cells	[208]
Anti-malarial drugs	/	Intercalate and bind to the minor groove of dsDNA that obstructs cGAS binding	Interact with the cGAS/dsDNA complex and effectively inhibit IFN production	[209]
Oligodeoxynucleotides (ODN) A151	5'-TTAGGGTTAGGGTTAGGGTTAGGG-3'	Mimic the ability of telomeric DNA, inhibitor of cGAS and AIM2 by competing with DNA	blocks cGAS-mediated type I IFN response induced by cytosolic DNA	[210, 211]
RU.521	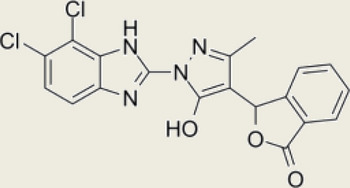	Block cGAS active center	Inhibitory effects on interferon stimulated by dsDNA in macrophage	[212]
RU.365 (Compound 7)	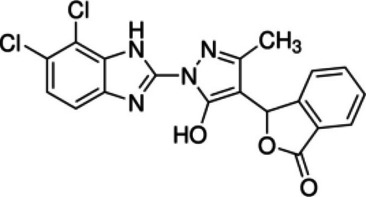	Reduce cGAMP by occupying the catalytic site of cGAS	Showed an IC_50_ value of 1.89 μM against cGAS	[212]
PF-06928215	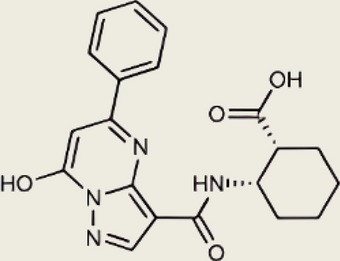	Interacted with Lys362 and Lys350 in cGAS	Inhibited the catalytic activity of cGAS by ATP and GTP, and interfered with the production of cGAMP	[213]
Suramin	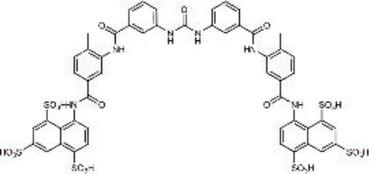	Displacing the bound DNA from cGAS	Regulates the production of IFN-β in THP-1 cells	[214]
I-a-9c	An isomer of nitrooleic acid	Inhibit cGAS through π-π stacking and π-cation interactions with cGAS's aromatic center; hydrogen bonding with Asp227	Compete with cGAS's natural substrates for binding to the catalytic site, thus inhibiting its enzymatic activity	[215]
CU-32, CU-76	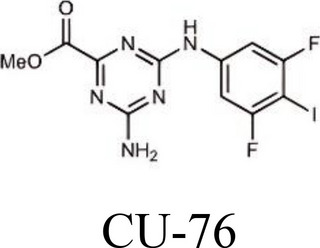	Target the DNA-binding site that displace the bound DNA from cGAS	The IC50 values of CU-32 and CU-76 in THP-1 cells were 0.66 and 0.27 μM	[85, 216]
Hydroxychloroquine	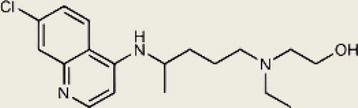	Inhibit cGAS by interfering with its binding to cytosolic DNA	Reduce the production of pro-inflammatory cytokines, including IFN-I	[217]
G150	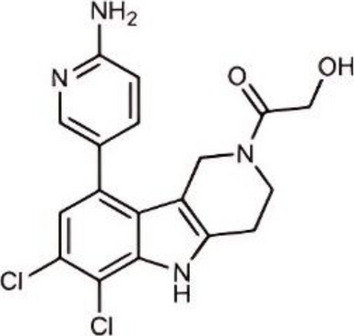	Catalytic site inhibitors	A novel small-molecule inhibitor that binds to cGAS and inhibits its activity	[218]
Quinacrine	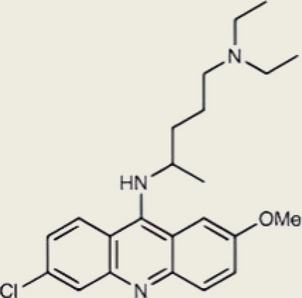	Binds to DNA directly	Attenuate cGAMP production in vitro and IFNβ expression in DNA‐stimulated THP1 cells	[219]
X6	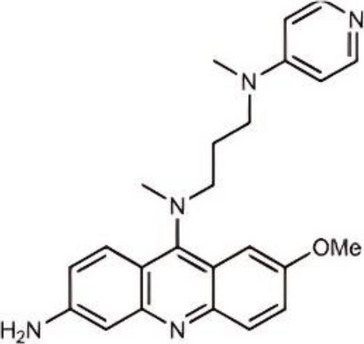	Inhibited cGAS activation competitively through dsDNA displacement from cGAS	Reduce the production of pro-inflammatory cytokines	[219]

**Table 2:** Representative STING inhibitors

STING inhibitors	Structure	Inhibition mechanism	Biological effect	Refs.
Tetrahydroisoquinoline	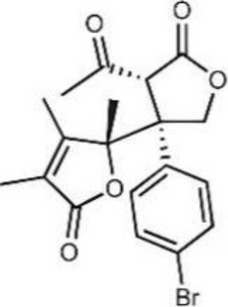	Binds to STING by replacing cGAMP	Inhibiting the cGAMP-dependent signaling pathway	[219]
Astin C	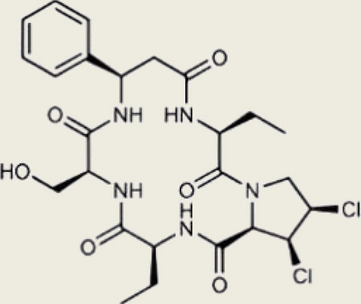	Forms a bond with the C-terminal binding region on STING proteins	Suppressing IRF3 gene expression without affecting TBK1 phosphorylation	[191]
C-176	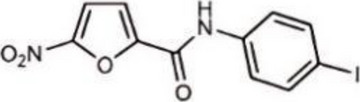	Target Cys91 on STING, blocking activation-induced palmitoylation	Displaying anti-inflammatory effects	[220]
C-178	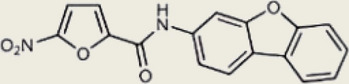	Target STING palmitoylation sites; form a covalent bond with TMD Cys91 and Cys88	Displaying anti-inflammatory effects	[220]
H-151	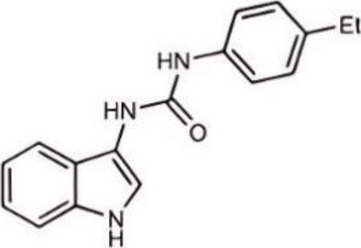	Targeting STING palmitoylation sites; It forms a covalent bond with TMD Cys91 and Cys88	Displaying anti-inflammatory effects	[220]
Nitro fatty acids	Fatty acids containing nitro (-NO₂) functional groups	Targeting STING palmitoylation sites; it forms covalent bonds with Cys88/91 and N-terminal His16	Anti-inflammation	[189]
Acrylamide	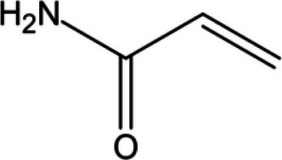	Targeting STING palmitoylation sites	Anti-inflammation	[221]
Compound 18	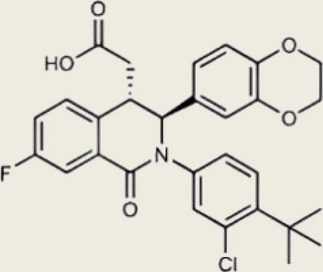	Hydrogen bonding is formed with Thr263 by carboxyl group	Induce IRF3-dependent antiviral effector genes, as well as type I and III IFN	[222]
C-170	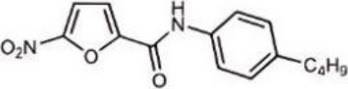	Alkylation of cysteine	Anti-inflammation	[223]
C-171	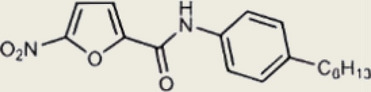	Alkylation of cysteine	Anti-inflammation	[223]
BPK-21	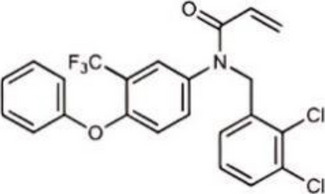	Associated with the Cys91 of STING, inhibiting the palmitoylation of STING	Inhibited STING activation in both THP1 and PBMC cells in response to cGAMP stimulation	[212]
BPK-25	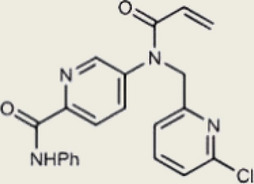	Associated with the Cys91 of STING, inhibiting the palmitoylation of STING	Inhibited STING activation in both THP1 and PBMC cells	[212]

Two major classes of cGAS inhibitors have been developed to block the synthesis of cGAMP. One class targets the catalytic active site of cGAS, whereas the other disrupts the interaction between cGAS and double-stranded DNA [224]. Small-molecule inhibitors such as RU.521 have demonstrated robust efficacy in suppressing cGAS signaling across multiple experimental models. RU.521 binds to the catalytic pocket of cGAS, effectively reducing cGAMP production and alleviating autoimmune phenotypes in Trex1-deficient murine models [225, 226]. More recently, VENT-03 has emerged as a first-in-class small-molecule cGAS inhibitor [227, 228]. By potently and selectively binding to cGAS, VENT-03 prevents the synthesis of cGAMP and subsequent downstream inflammatory cascades. Given its excellent preclinical safety and efficacy profile, VENT-03 has recently entered early-phase clinical trials targeting SLE and other cGAS-driven autoinflammatory diseases, marking a significant milestone in cGAS-targeted therapeutics. Additionally, modifying liquid–liquid phase separation represents an alternative inhibitory strategy. For instance, the phase separation inhibitor A151 competitively blocks cytosolic dsDNA binding [211, 229], and oleic acid-based nanocarriers have been shown to dissolve pathological cGAS-DNA condensates to suppress aberrant immunity.

For STING inhibition, regulation can be achieved by targeting its ligand-binding domain (LBD) or specific post-translational modifications (such as palmitoylation at Cys88/91) [230]. Compounds like H-151, nitrofuran, and indole urea derivatives covalently bind to STING, blocking its activation-induced palmitoylation and subsequent oligomerization [231, 232]. Recently, SN-011 has been developed as a highly potent and selective STING antagonist [233]. SN-011 specifically binds to the cyclic dinucleotide-binding pocket of STING, locking it in an inactive open conformation. Pharmacological evaluation indicates that SN-011 broadly and profoundly suppresses systemic inflammation, rescues disease phenotypes in AGS mouse models (Trex1^−/−^), and significantly decreases mortality rates, highlighting its tremendous potential for clinical translation in severe autoimmune diseases [234].

### Biologic and gene-targeted therapies

While small-molecule inhibitors dominate current developmental pipelines due to their intracellular penetrability, biologic and gene-interference technologies offer highly specific alternative approaches to modulate the cGAS-STING pathway. Antibody-based therapeutics have emerged as an active area of investigation [235]. Specific antibodies targeting STING can effectively neutralize its activity and mitigate pathological inflammation. Owing to their high target specificity and relatively favorable safety profiles, STING-neutralizing antibodies are being explored as valuable therapeutics for systemic autoimmune diseases, minimizing the off-target toxicity frequently associated with small molecules.

Gene-interference strategies represent another highly precise approach. Gene-silencing tools, such as small-interfering RNAs (siRNAs) and antisense oligonucleotides (ASOs), can directly suppress the expression of cGAS or STING at the mRNA level, thereby dampening aberrant pathway activation [226]. siRNA-based approaches have demonstrated favorable safety profiles in preclinical models and have shown promise in attenuating chronic neuroinflammation and autoimmune responses by directly reducing the cellular pool of cGAS/STING sensors.

### Agonists and advanced delivery systems

In cancer immunotherapy, activation of the cGAS-STING pathway is highly desirable to prime tumor-specific CD8 + T cells and reprogram the immunosuppressive TME [236]. Cyclic dinucleotides (CDNs), functioning as natural STING agonists, have been widely explored [237]. However, natural CDNs face major clinical hurdles, including poor membrane permeability, metabolic instability, and rapid systemic clearance [238].

To overcome these barriers, novel synthetic agonists and delivery systems have been rapidly developed. IMSA101 is a representative synthetic CDN STING agonist formulated for intratumoral injection [239]. By binding to STING, IMSA101 induces robust IFN-beta production, promotes cross-presentation by dendritic cells, and enhances tumor-infiltrating lymphocyte proliferation. It is currently undergoing extensive clinical evaluation for solid tumors. Concurrently, non-nucleotide small-molecule agonists, such as IMSB301, have been engineered to overcome the pharmacokinetic limitations of CDNs [240]. IMSB301 demonstrates excellent metabolic stability and membrane permeability, enabling systemic (intravenous or oral) administration, which is crucial for treating metastatic cancers that are inaccessible to direct intratumoral injection.

Furthermore, advanced nanotechnology-based delivery approaches have shown great promise [241–243]. Polymer-based nanoparticles and lipid-based carriers can encapsulate CDNs or small-molecule agonists, promoting their targeted accumulation within the TME while reducing systemic cytokine storms [244]. For example, specific bio-mineralized outer membrane vesicles [182] and engineered tumor-homing bacteria [29] have been utilized to release STING agonists locally, triggering robust and precise immune activation within the tumor bed while sparing healthy tissues.

### Targeting downstream effectors: TBK1 and JAK inhibitors

In addition to directly targeting cGAS or STING, inhibiting downstream effectors offers a critical and clinically validated alternative, particularly for patients with hyperactive interferonopathies. The primary downstream kinases in the cGAS-STING axis include TBK1 and the Janus Kinases (JAKs), which mediate IRF3 phosphorylation and IFN receptor signaling, respectively [104, 245].

TBK1 inhibitors (such as GSK8612 and amlexanox) selectively block the phosphorylation of IRF3, effectively severing the signaling cascade between STING activation and interferon gene transcription [246, 247]. In preclinical models of ALS and TREX1-mediated autoimmune diseases, TBK1 inhibition has shown significant efficacy in halting neurodegeneration and systemic inflammation. More importantly, JAK inhibitors (such as Tofacitinib, Baricitinib, and Ruxolitinib) block the downstream IFNAR/JAK/STAT signaling cascade that is excessively triggered by STING activation. Because JAK inhibitors are already FDA-approved for indications like rheumatoid arthritis and psoriasis, they have been successfully repurposed for cGAS-STING-driven monogenic autoinflammatory diseases [248]. For instance, Baricitinib has demonstrated life-saving efficacy in clinical settings for patients with STING-associated vasculopathy with onset in infancy and Aicardi-Goutières syndrome (AGS) [249, 250], successfully alleviating severe systemic inflammation and tissue damage where direct STING inhibitors are still pending clinical availability.

### Combination strategies and clinical advances

The translation of cGAS-STING targeted therapies from bench to bedside has yielded both promising breakthroughs and significant learning curves. While preclinical models demonstrated profound tumor regression with STING agonist monotherapy, early clinical trials (such as those involving ADU-S100) revealed limited single-agent efficacy [251]. This was partly attributed to systemic toxicity at high doses and T-cell apoptosis triggered by hyper-activated STING. Consequently, the clinical paradigm has shifted heavily toward combination strategies [206].

Current clinical trials emphasize combining STING agonists with immune checkpoint inhibitors (ICIs) or genotoxic therapies to achieve synergistic antitumor immunity. For instance, in a Phase 1/2 clinical trial (NCT04020185), the synthetic STING agonist IMSA101 is being evaluated in combination with Pembrolizumab (an anti-PD-1 antibody) for patients with advanced solid tumors [239, 252]. The rationale is that IMSA101 converts immunologically cold tumors into hot ones by driving dendritic cell maturation and T-cell infiltration, thereby overcoming primary resistance to PD-1 blockade.

Radiotherapy also synergizes exceptionally well with the cGAS-STING pathway [253, 254]. Radiation-induced DNA damage leads to the release of fragmented DNA into the cytosol, intrinsically activating cGAS. When combined with exogenous STING agonists or ICIs, this radiation-primed cGAS activation triggers an amplified systemic immune response, known as the abscopal effect, which aids in eradicating distant metastases. Table 3 summarizes the representative cGAS-STING targeted drugs currently under clinical investigation, detailing their mechanisms, target indications, and clinical trial status.

**Table 3:** Representative clinical trials of therapeutics targeting the cGAS-STING pathway and downstream effectors

Drug name	Target and mechanism	Primary indication	Clinical trial no	Phase	Current status
VENT-03	cGAS inhibitor	Systemic Lupus Erythematosus	NCT07260877	Phase 2a	Recruiting
IMSA101	STING agonist	Refractory Malignancies	NCT04020185	Phase 1/2a	Completed
ADU-S100 (MIW815)	STING agonist	Advanced Solid Tumors/Lymphomas	NCT02675439	Phase 1	Terminated
MK-1454	STING agonist	Head and Neck Squamous Cell Carcinoma	NCT04220866	Phase 2	Completed
E7766	STING agonist	Advanced Solid Tumors/Lymphoma	NCT04144140	Phase 1	Terminated
TAK-676	STING agonist	non-small-cell lung cancer, triple-negative breast cancer and squamous-cell carcinoma of the head and neck	NCT04879849	Phase 1	Completed
Baricitinib	Downstream Inhibitor of JAK1/JAK2	Rheumatoid Arthritis	NCT03922769	Phase 2	Completed
Tofacitinib	Downstream Inhibitor of JAK1/JAK3	Juvenile Idiopathic Arthritis	NCT01500551	Phase 2/3	Completed

Data derived from ClinicalTrials.gov. on April 27, 2026

## Conclusion

The cGAS–STING signaling pathway, a central mechanism for intracellular DNA sensing, has emerged as a critical regulator of innate immunity. As highlighted throughout this review, its spatiotemporal regulation, which is governed by phase separation, organelle dynamics, and metabolic cross-talk, reveals unprecedented layers of complexity that shape immune outcomes across diverse pathologies. The pathway exhibits a profound context-dependent duality. Specifically, acute physiological activation robustly drives antimicrobial defense and antitumor immunity; conversely, chronic signaling paradoxically fuels immunosuppression, metabolic dysregulation, and severe autoimmune inflammation.

The integration of emerging evidence from structural biology, nanotechnology, and clinical studies has enabled a paradigm shift in therapeutic development, yet significant translational challenges remain. A primary hurdle in current clinical applications is achieving precise tissue-specific targeting. Because systemic modulation of the cGAS-STING axis can readily provoke adverse off-target effects (including potentially fatal cytokine storms with systemic agonists or profound immunosuppression with systemic inhibitors), precision targeting is paramount. While recent successes, such as advanced nanoparticles amplifying STING activation specifically within TME, highlight the promise of microenvironment-responsive delivery systems, developing highly specific nanocarriers and targeted conjugates to maximize localized efficacy while minimizing systemic toxicity remains a formidable task for future drug design.

Looking ahead, several key research directions will accelerate progress toward individualized therapies over the next few years. First, unraveling the pathway's role in the Senescence-Associated Secretory Phenotype is critical. Emerging evidence indicates that chronic cGAS-STING activation, triggered by genomic or mitochondrial DNA leakage in senescent cells, is a fundamental driver of SASP and chronic neuroinflammation, presenting a highly promising therapeutic target for age-related and neurodegenerative diseases. Second, deeper exploration into the non-canonical functions of STING is urgently needed. Future studies must look beyond traditional interferon-centric paradigms to fully elucidate STING’s independent roles, particularly its newly discovered function as a proton channel and its regulation of non-canonical autophagy and lysosomal homeostasis. Third, advancing translational tools and validating clinical biomarkers, such as establishing cGAMP or cell-free dsDNA levels as quantifiable indicators, will be essential for optimizing patient stratification and monitoring treatment efficacy.

Ultimately, the cGAS–STING pathway exemplifies the convergence of mechanistic discovery and therapeutic innovation. By bridging fundamental biological insights with cutting-edge targeted technologies, we are poised to transform this signaling axis into a cornerstone of precision medicine across oncology, autoimmune disorders, and neurodegeneration. Future breakthroughs will undoubtedly hinge on interdisciplinary collaboration and a steadfast commitment to contextualizing pathway biology within the full spectrum of human disease.

## Data Availability

Not applicable.
